# Cultural Foundations of the Second Demographic Transition: The Role of Inherited Values

**DOI:** 10.1007/s10680-025-09759-1

**Published:** 2025-12-08

**Authors:** Hande Tugrul, Arnstein Aassve

**Affiliations:** 1 Department of Social and Political Sciences, Bocconi University, 4 Via Roentgen, 20136 Milano, Italy; 2 Department of Social and Political Science, Carlo F. Dondena Centre for Research on Social Dynamics and Public Policy, Bocconi University, 4 Via Roentgen, 20136 Milano, Italy

**Keywords:** Second demographic transition, Inherited values, Non-marital childbearing, Family formation, Culture

## Abstract

Considerable variation exists across societies in the prevalence of demographic trends associated with the second demographic transition (SDT). We propose that these persistent disparities are, in part, determined by long-standing cultural traits. Employing an epidemiological approach, we proxy the inherited component of five key values—gender egalitarianism, religiosity, institutional distrust, generalized trust, and family ties—from the descendants of immigrants in the United States, and link them to SDT outcomes across 23 countries. Our analysis investigates whether societies pre-exposed to these specific values through intergenerational transmission are more or less likely to exhibit SDT, operationalized here as the share of births outside marriage. Our findings reveal that several of these traits exert a notable influence when interacting with educational expansion. Gender egalitarianism, institutional distrust, and generalized trust exhibit positive associations with non-marital birth rates when coupled with increased education. Meaning that, with the broad educational expansion that has taken place across all Western countries after the IIWW, the SDT spreads much faster in societies where these three inherited values are deeply ingrained. Conversely, family ties demonstrate a negative association, while no strong evidence is found regarding the influence of religiosity. In conclusion, our study underscores the necessity of a nuanced cultural approach to the SDT framework, acknowledging the importance of local values alongside the global ideational shift.

## Introduction

In the second half of the 20th century, Western societies bore witness to a profound transformation in family formation and fertility behaviours. Led on by the Nordic countries, a notable shift occurred, challenging the traditional centrality of the family. It was accompanied by liberal demographic behaviours such as cohabitation, non-marital childbearing, divorce, and a postponement of marriage and parenthood. The second demographic transition (SDT) theory suggested that societies were navigating successive stages of global change, with a pivotal role played by an ideational shift. The increased material security provided by advanced post-war economies facilitated the emergence of “higher-order” (Maslow, 1954) needs and “post-materialist” (Inglehart, 1977) values, which prioritized self-realization, freedom of expression, and autonomy over conformity to societal expectations. This ideational shift, along with subsequent structural changes such as educational expansion, leads individuals to postpone long-term commitments like marriage and parenthood.

**Fig. 1:**
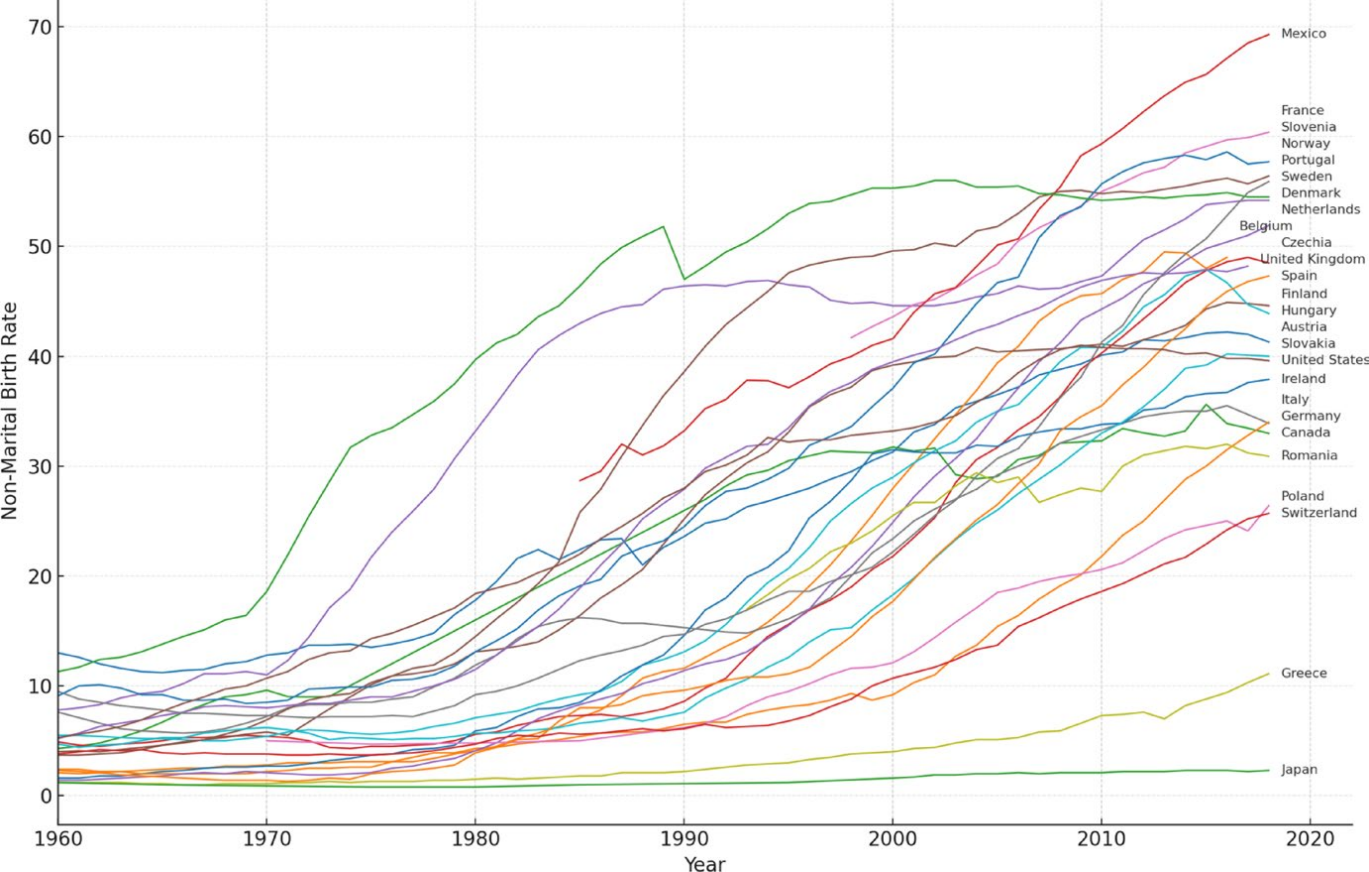
Share of births outside marriage. Proportion (%) of all births where the mother’s marital status at the time of birth is other than married. Data retrieved from OECD Family Database

Though Western countries experience a shared value shift to some extent, the onset and intensity of the increase in demographic trends typically associated with varied widely across nations (Fig. 1). There is consequently still a lively debate about the capacity of the SDT framework to provide a theoretical explanation of contemporary demographic trends, specifically the differences across societies rather than similarities (Zaidi & Morgan, 2017). A fundamental question arises: to what extent are these new demographic behaviours determined by the diffusion of profound changes in value orientations?

In contrast to its great emphasis on cultural change, the SDT framework pays less attention to the impact of a society’s long-standing cultural history on its demographic trends. While it acknowledges the influence of local culture as a path dependency modulating the speed and intensity of SDT’s spread (Lesthaeghe, 2010, 2020), it falls short of delving into how specific cultural traits impact recent demographic trends. The critique offered in this article is not to refute the global cultural shift proposed by the SDT theory but rather to provide a fine-tuned cultural approach by drawing attention to the role of a society’s long-standing values inherited across generations. Putting it differently, the aim is to investigate whether societies pre-exposed to specific values due to intergenerational transmission of culture are more likely to be the leaders of SDT. Hence, we pose two fundamental questions: What role does a society’s longstanding culture play in the de-standardization of family and fertility dynamics associated with the SDT? Specifically, which values are transmitted across generations that, in interaction with educational expansion, have significance for the SDT? Empirically, we focus on the share of births outside marriage as our SDT indicator. We privilege this outcome because it combines historical depth and cross-national comparability with a close conceptual link to the SDT’s ideational core—marriage becoming less of a prerequisite for childbearing.

In this regard, the article offers three significant contributions. Firstly, it extends the understanding regarding the cultural foundations of the demographic change witnessed in the last decades. It identifies and empirically tests a list of inherited values, including gender egalitarianism, religiosity, institutional distrust, generalized trust, and family ties, that yield motivation for de-standardizing family and fertility dynamics.

Secondly, we focus on an often-neglected component of culture, namely the inherited values, and follow their variation over extended periods. We derive measures of inherited values by adopting Algan and Cahuc’s take on epidemiological approach (2010). Rather than relying on reported values from the World Values Survey or similar sources—which inevitably reflect the contemporary conditions of the societies in which respondents live—the epidemiological approach extracts values from U.S. respondents with ancestral origins abroad. These responses provide proxy measures of inherited values in the corresponding country of origin. Importantly, while the U.S. General Social Survey supplies the data infrastructure for this step, our analysis is cross-country in focus, examining demographic outcomes in 23 origin countries.

Third, this methodological design enables us to revisit the cultural foundations of SDT. The SDT framework emphasizes a global ideational shift as the main driver of demographic change. By focusing instead on the enduring influence of specific inherited cultural traits, we demonstrate how long-standing values condition the spread of SDT. More precisely, it establishes the extent to which particular long-standing cultural traits foster or impede the demographic outcomes of SDT as societies are experiencing important structural changes such as female education expansion.

## Background

### Can Long-Standing Cultures Explain the Growing Divergence in Second Demographic Transition?

From the late 1960s onwards, the demographic trends in industrialized societies deviated from the predictions of the first demographic transition. Specifically, in Western and Northern European societies, declining fertility did not stop at the replacement level, the popularity of the traditional family decreased among young cohorts, leading to the emergence of alternative living arrangements and the disconnection of marriage and childbearing. Lesthaeghe and Van de Kaa introduced the second demographic transition theory as an attempt to explain these radical changes in fertility and family formation (Lesthaeghe & Van de Kaa, 1986; Kaa, 1987). Aligning with Inglehart’s argument on post-materialism (1977, 1990), the theory took ideational factors and culture change at centre stage by arguing that industrialized societies entered into a profound shift in value orientations toward individualization, self-actualization, and secularization during the postwar period (Lesthaeghe, 2010; van de Kaa, 1987; Kaa, 2002).

The cultural transmission of the new values across individuals is governed by educational expansion. Education, particularly female educational attainment, can be perceived as a pr for cultural endowment, which is linked to non-conformism, greater emphasis on self-fulfilment, individualization, sexual liberation, and higher tolerance to unconventional behaviours. These factors collectively reshape family and fertility dynamics in society (Lesthaeghe & Surkyn, 1988). In this framework, female education expansion is not just a predictor of SDT behaviours, but a moderator that amplifies or attenuates the influence of inherited cultural values on family formation. For instance, when education levels rise, values such as gender egalitarianism and generalized trust become more salient in shaping non-marital childbearing behaviours. In societies with higher female educational attainment, these cultural values can drive an increase in non-marital births, as education facilitates the decoupling of marriage and childbearing. This interaction aligns with Aassve et al. (2016), which highlights education as a structural factor that interacts with cultural values to influence demographic outcomes like fertility and family formation.

The SDT framework emphasizes a broad post-war ideational shift toward individual autonomy, but anticipates lasting cross-national heterogeneity rooted in historical and cultural path dependence (Lesthaeghe, 1994, 2011, 2014; Lesthaeghe & Surkyn, 2002, 2004; van de Kaa, 2002; Kaa, 1994, 2001). Empirically, countries differ in the timing, pace, and saturation levels of SDT markers (cohabitation, non-marital childbearing, divorce, very low fertility), with divergence documented across and within Europe (Billari & Liefbroer, 2010; Billari & Wilson, 2001; Kuijsten, 1996; Zaidi & Morgan, 2017).

The theory claims that the local culture modulates the connection between the adoption of ideational change and its reflection on demographic behaviour. Thus, the cross-country differences in SDT’s emergence and speed are contingent on historical path dependencies (Lesthaeghe, 2010, 2020). This perspective is not wrong but remains limited in scope since it backgrounds society’s long-standing culture and thus fails to answer how and which cultural traits encourage or discourage demographic change.

Adopting a historical perspective, Reher (1998, 2004) emphasises the importance of enduring cultural factors in influencing contemporary fertility and family behaviours. He underscores that certain cultural values persist across generations, shaping individuals’ choices and behaviours. This persistence is particularly relevant in the context of SDT since traditions related to family, gender roles, and marriage often have deep historical roots and resist rapid change, even in the face of broader societal transformations. Recognising cultural persistence helps explain why some societies experience demographic transitions more gradually or with greater resistance and underscores the need for culturally sensitive approaches to demographic behaviour.

### Which Inherited Cultural Traits?

Recent sociological and anthropological accounts conceptualise culture as multi‑layered, with one stratum rooted in long‑term historical processes transmitted intergenerationally and another more exposed to contemporaneous developments and modernisation (Bachrach, 2014; Guetto, 2012). Behavioural change typically begins in the latter stratum but remains conditioned by the former: when individuals encounter cultural developments in their social environments, they reinterpret them according to long‑standing cultural models.

Intending to answer these questions, we distinguish two constitutive elements of culture—models and values (Patterson, 2014). Models capture shared schemas and practices that provide predictability and continuity to social action and interaction, whereas values represent the evaluative dimension at the micro‑level, referring to individuals’ prioritizations, and preferences, thus reflecting the desirability of means and ends of actions. Prior research indicates that values are persistent and transmitted across generations to different extents (Glass et al., 1986; Rohan & Zanna, 1996). Building on the intergenerational transmission literature, value similarity between parents and offspring is sustained through socialization, modelling, and normative sanctions within families and kin networks (Bengtson et al., 2009). Following Rohan and Zanna (1996), we treat values as relatively enduring, abstract guides to action—allowing partial but non-trivial persistence across generations rather than assuming perfect stability.

In this context, we anticipate that specific long-standing inherited values, when coupled with the expansion of education, will have a significant impact on the evolution of SDT in a given country – specifically the share of births outside marriage. We begin from Lesthaeghe and Surkyn’s (1988, 2004) list of values typically associated with demographic characteristics of SDT, and then we leveraged insights from the extensive literature on family and fertility. Data limitations preclude including all potentially relevant values; consequently, we focus on five inherited values: gender egalitarianism, religiosity, institutional distrust, generalized trust and family ties.

#### Gender Egalitarianism

The original theory and its extensions encompass the concept of the gender revolution, which includes a growing symmetry in gender roles and female economic autonomy, as integral components of the societal context for SDT (Lesthaeghe, 2020). While it alone cannot explain demographic trends, it is a vital part of the multifaceted revolution that catalysed SDT (Lesthaeghe, 2010).

Furthermore, Lesthaeghe and Surkyn identify gender egalitarianism as one of the values typically associated with SDT’s demographic trends (1988, 2004). The ideational shift towards individualization, rejection of authority, and self-actualization manifests in gender dynamics, with a desire for equal gender roles in both public and private spheres (Lesthaeghe & Neels, 2002). In conjunction with critical structural developments such expanded higher education and greater access to birth control methods, these value changes delayed women’s family transitions, altered their perspectives on parenthood, and reduced the preferred number of children. Moreover, as these shifts have propelled the dual-earner model, gender-egalitarian relations have become a factor influencing the quality of unions. In the cases where certain quality is not met, divorce and single parenthood have become viable options (Lesthaeghe & Neels, 2002). Against this backdrop, we propose the following hypothesis:

##### Hypothesis 1

Countries with higher inherited gender egalitarianism will demonstrate higher share of births outside marriage (our SDT indicator) as female education expands, reflecting the reduced necessity of marriage as a prerequisite for childbearing.

#### Religiosity

Previous works has posited a reciprocal relationship between secularization and non-conventional family formation, where each can mutually reinforce the other (Thornton, 1985; Thornton et al., 1992). These studies demonstrated that greater secularism promoted non-conventional unions and sexual behaviours, subsequently advancing secularism further. In a similar vein, the SDT framework identifies secularism as one of the value shifts associated with the de-standardization of family life and fertility patterns (Lesthaeghe & Surkyn, 1988, 2004). Subsequent empirical investigations have supported this premise (Lesthaeghe, 2010; Lesthaeghe & Neels, 2002; Lesthaeghe & Surkyn, 2004; Moors, 2008).

On an individual level, secularism denotes the abandonment of religiosity, entailing a decline in spiritual sentiments, traditional religious beliefs, and practices. Also, previous studies have shown it is a persistent social product shaped within the family during the early years of socialization; therefore, as a value, it can be strongly transmitted across generations (Bengtson et al., 2009; Myers, 1996). Given these, we suggest the following hypothesis:

##### Hypothesis 2

Countries with higher levels of inherited religiosity will demonstrate lower share of births outside marriage as female education expands, given religion’s emphasis on marriage as the normative context for childbearing.

#### Institutional Distrust

A fundamental element of the cultural shift that underpins the SDT theory is the preference for individual autonomy over any form of institutional authority. Lesthaeghe and Surkyn (2004) contend that this rejection of authority is mirrored in the political field as the value of the “new political left” and list it under the initial set of values related to SDT behavior. The new political left encompasses several dimensions linked to Inglehardt’s (1977) postmaterialism, yet due to data limitations, we focus solely on distrust in institutions.

We posit that established institutions tend to promote conformity to conventional forms of conduct in various domains, including gender roles, family, and fertility. Consequently, their rejection is likely to reinforce the transformation of gender roles and the emergence of diverse family structures. Also, if the family is considered the smallest and most ancient social institution, rejecting its institutional foundations is likely to bring flexibility to union formation and dissolution. In light of these, we propose the following hypothesis:

##### Hypothesis 3

Countries with lower inherited trust in institutions will demonstrate higher share of births outside marriage as female education expands, consistent with weaker adherence to institutionalized life-course scripts.

#### Generalized Trust

Generalized trust, which measures an individual’s trust in those outside their immediate circle, forms a crucial aspect of societal cohesion and cooperation (Aassve et al., 2016). It fosters a sense of community and eases interactions, offering a safety net. While generalized trust may vary in the short term based on individual experiences and morals, its long-term stability in society is well-documented (Uslaner, 2002). with persistent disparities among different societies (Bjørnskov, 2007).

Although the SDT theory doesn’t explicitly reference generalized trust, prior research indicates its substantial impact on reproductive dynamics in industrialized societies (Aassve et al., 2016, 2021). These studies argue that the impact of generalized trust on fertility operates through two main channels. Firstly, high generalized trust correlates with positive societal outcomes, including effective institutions, political engagement, social cohesion, economic growth, lower corruption, and reduced crime rates—all conducive to a stable environment for child-rearing. Secondly, generalized trust encourages individuals to outsource childcare, which is especially important as women pursue higher education and careers that may not easily align with childrearing (Aassve et al., 2016).

In the context of generalized trust and SDT, we posit that heightened trust levels may reduce the appeal of traditional marriage, leading to a shift in perceptions from marriage as a secure, legally regulated institution to viewing voluntary singlehood, union dissolution as viable alternatives. Furthermore, as generalized trust facilitates the delegation of childcare responsibilities, individuals may be more inclined to postpone childbearing to later stages of life. Indeed, it was found that countries that show more characteristics of SDT also have high levels of generalized trust (Aassve et al., 2013). Given these, we suggest the following hypothesis:

##### Hypothesis 4

Countries with higher inherited generalized trust will exhibit higher share of births outside marriage as female education expands, since trust lowers perceived risks and makes alternative family forms more viable.

#### Family Ties

Since all behavioural outcomes of SDT point towards the liberation of family, the strength of family ties reflecting the importance of family can be seen as a straightforward factor. Societies exhibit substantial variation in the prevalence of close and weak ties among family members. Reher (1998) characterizes this distinction as the historically “strong family systems” found in Southern Europe and the “weak family systems” prevalent in Western and Northern Europe.

Within the weak family system, children typically leave their parental homes before marriage and enter an interim phase where they may choose to live independently, share accommodations, or cohabit with a partner. This period is likely to be extended in regions with generous welfare provisions and extension of education. Additionally, the weak family system aligns with values promoting gender equity, individualization, and self-expression, making it compatible with the principles of SDT.

Contrarily, in the strong family systems, individuals tend to reside at parental homes until marriage. Welfare provisions addressed to single individuals or students are relatively limited; thus, necessitating support from their parents, young adults become economically independent much later in life. This model tends to sustain traditional gender roles, lead to earlier marriages and childbirth, and reduce the prevalence of practices like cohabitation and non-marital childbearing. Lesthaeghe (2010) has also drawn upon this theory to explain the lag experienced by Southern European countries in the context of SDT. In this regard, we propose the following hypothesis:

##### Hypothesis 5

Countries with stronger inherited family ties will demonstrate lower share of births outside marriage as female education expands, reflecting persistent expectations about intergenerational support and sequencing.

## Analytical Strategy

A significant empirical challenge in assessing the influence of inherited values on SDT lies in the scarcity of standardized and geographically widespread data on values from earlier periods. Key databases used for measuring values, such as The World Values Survey and European Social Survey, were initiated after the 1980s. Since we cannot directly observe the values of previous generations, we adopt Algan and Cahuc (2010)’s take on epidemiological approach to proxy the inherited components of culture across countries. Accordingly, our present-day values result from two primary factors: values inherited through intergenerational transmission and the contemporary environment. Therefore, to differentiate the inherited components from the influence of the contemporary environment, they proxy country-specific cultural legacies using the values of U.S. residents who are descendants of immigrants, and then assign these values back to their countries of origin (Algan & Cahuc, 2010).

This approach allows us to reconstruct historical value patterns in contexts where no direct data exist and it controls for confounding effects of the contemporary environment. By analysing descendants of migrants within a shared host context (the U.S.), we recover origin-country proxies for inherited values; the U.S. survey functions as a shared data infrastructure rather than the object of inference. We acknowledge that, the U.S. as a host context is not culturally uniform - local ethnic enclaves, parish networks, or region-specific norms could shape reported values even among later-generation respondents. To limit such contextual contamination, we exclude first-generation immigrants and control for many individual characteristics including respondent generation (2nd /3rd /4th ), U.S. Census region etc. and we average across dispersed respondents and cohorts. This controlled design minimizes distortions from both origin- and host- countries, enabling us to capture the long-term, transmitted component of values and to clarify how intergenerational value transmission persists or changes over time.

However, the epidemiological approach is not without its limitations. Focusing on migrants’ descendants may not accurately represent the broader population, thereby introducing the risk of selection bias. Migrant groups often have unique characteristics or undergo historical experiences that differentiate them from non-migrant groups or they may come from varied socio-economic and cultural backgrounds (e.g. economic migrants from rural areas or political migrants). The approach attempts to mitigate these by controlling for a wide range of socio-economic and demographic variables, thereby ensuring that the inherited values observed are not unduly influenced by the unique characteristics or experiences of migrants.

Overall, our analytical strategy consists of two stages. In the first stage, we proxy the inherited values of people living in country c by using the values that descendants of U.S. immigrants have inherited from their ancestors who have migrated from country c. While the survey respondents reside in the U.S., the proxied cultural measures are assigned back to their ancestral countries. Once we obtain country-level inherited values for two different years sufficiently apart, we use them in the second stage, where we perform the cross-country macro-level analysis outlined in our linear model (1).

### First Stage: Individual Level Data and Estimation of Inherited Values

The estimation of inherited values is based on the epidemiological approach (Algan & Cahuc, 2010). This approach relies on the premise that value formation is shaped by two significant influences: the contemporary environment and inheritance from earlier generations (Benabou & Tirole, 2006; Bisin et al., 2004; Bisin & Verdier, 2001; Tabellini, 2008, as cited in Algan & Cahuc, 2007). Therefore, in cases where we don’t have access to previous cohorts’ reported values, we can proxy the inherited culture by isolating it from the contemporary environment. To achieve this, we exploit the intergenerational cultural transmission path across immigration cohorts, under the assumption that inherited values are not immediately overdetermined by the current characteristics of the country in which individuals reside (Algan & Cahuc, 2010).

We gather data on individuals’ values from the US General Social Survey (GSS) (Davern et al., 2022), a database offering rich information on specific values, birthplace, and ancestorial country of origin. In the GSS, respondents identify their ancestral country, allowing us to recover the cultural legacies of their origin countries. Here, the U.S. serves solely as a neutral host environment, ensuring that inherited values reflect intergenerational transmission rather than recent developments in the origin countries. Moreover, questions about the birthplace of respondents, as well as their parents and grandparents, enable us to identify four immigration waves: fourth-generation Americans (more than two grandparents born in the US and both parents born in the US), third-generation Americans (at least two grandparents foreign-born, and both parents were born in the United States), second-generation Americans (at least one parent born abroad) and first-generation Americans. We exclude first-generation Americans from our analysis because they are personally exposed to their country of origin. Such exposure to the contemporary demographic trends of the origin country may introduce endogeneity concerns.

Although the GSS begins in 1972, we approximate values for the 1960 anchor by drawing on older cohorts observed in the earliest GSS waves, whose formative years correspond to the 1950–1960 s. This allows us to reconstruct inherited values at the earlier anchor despite the absence of direct survey data. Our design is therefore period-anchored (1960 vs. 2010), rather than cohort-tracked. Inherited values are then linked to countries of origin through the U.S. immigrant sample, providing the cultural indicators for Step 2. This procedure mirrors prior epidemiological application of Algan and Cahuc (2010), where earlier-period beliefs are proxied through immigrant cohort timing.

To further minimize the possibility of endogeneity, we impose a lag of 25 years between value measurement and the observation of SDT outcome, namely non-marital birth rates. Conceptually, this lag reflects one full generation, ensuring that the inherited values used are formed sufficiently prior to the demographic behavior they are intended to predict. Put differently, for year T we use values from individuals whose generational positions imply value formation at least 25 years earlier: second-generation Americans born before T – 25, third-generation Americans born before T − 25 + 25, and fourth-generation Americans born before T − 25 + 50.

We apply this estimation of values to two distant years, 1960 and 2010. The 50 years of distancing between these time points is important for ensuring that immigration cohorts do not overlap significantly, the evolution of values over time is substantive, and does not comprise measurement errors. Following the described procedure, the values for 1960 correspond to the values of the second-generation Americans born before 1935, the third-generation Americans born before 1960, and the fourth-generation Americans born before 1985. Likewise, the values for 2010 are composed of the values of second-generation Americans born after 1935, third-generation Americans born after 1960, and fourth-generation Americans born after 1985. The distribution of the GSS sample of the second, third, and fourth generations is shown in Table 1.

**Table 1:** Number of respondents by origin country, inherited values, and year

	Gender egalitarianism		Religiosity		Institutional distrust		Generalised trust		Family ties	
	1960	2010	1960	2010	1960	2010	1960	2010	1960	2010
Austria	42	3	25	6	94	11	85	12	82	13
Belgium	12	1	16	2	29	3	26	4	36	3
Canada	148	20	100	38	267	51	282	58	276	57
Czechoslovakia	110	16	87	22	215	37	232	37	226	41
Denmark	64	6	46	13	135	14	131	17	141	14
Finland	24	4	27	6	92	10	82	13	86	14
France	199	11	154	26	377	42	385	49	392	44
Germany	1630	76	1280	215	3368	280	3504	305	3395	296
Greece	17	13	22	16	46	26	45	32	46	33
Hungary	36	5	28	10	81	15	77	21	79	22
Ireland	1121	55	1115	154	2429	198	2595	216	2500	223
Italy	416	90	333	190	812	302	880	325	874	304
Japan	8	3	8	4	25	10	28	12	23	16
Mexico	124	79	112	207	236	274	276	305	246	327
Netherlands	149	11	98	16	261	30	273	32	263	38
Norway	187	6	133	21	324	32	355	32	349	32
Poland	234	36	174	65	469	109	471	115	480	114
Portugal	14	3	18	11	36	20	43	20	41	19
Romania	7	3	7	3	15	3	14	8	16	6
Spain	53	7	69	31	121	41	133	42	114	34
Sweden	152	9	132	21	330	28	335	33	329	34
Switzerland	36	2	44	2	88	3	95	4	89	3
United Kingdom	1639	50	1364	113	3332	169	3480	175	3413	150
United States	183	2	267	29	520	34	565	37	553	34
Yugoslavia	33	5	19	9	59	19	60	20	71	20

Data is from authors’ calculation from US General Social Survey Sample

We based our measurement of each value on individuals’ answers to related questions in the GSS.

Firstly, to capture various dimensions of gender role orientations, we composed a gender egalitarianism indicator using four questions: *“A working mother can establish just as warm and secure a relationship with her children as a mother who does not work*,*” “It is more important for a wife to help her husband’s career than to have one herself*,*” “A preschool child is likely to suffer if his or her mother works*,*” “It is much better for everyone involved if the man is the achiever outside the home and the woman takes care of the home and family.”* We recoded answers to all statements such that higher values correspond to greater gender egalitarianism. Later, we used principal component analysis to construct a standardized index.

Religiosity is a multi-dimensional phenomenon, including individual beliefs and institutional foundations (Guetto et al., 2015). Thus, we measured it with a composite index generated using four questions. While we captured the beliefs dimension by the questions *“Would you call yourself a strong (preference named in religion) or a not very strong (preference named in religion)?”* and *“Please look at this card and tell me which statement comes closest to expressing what you believe about God”*, we included the institutional dimension with the questions *“How often do you attend religious services?”* and *“As far as the people running these institutions are concerned*,* would you say you have a great deal of confidence*,* only some confidence*,* or hardly any confidence at all in organized religion?”* Then, we recoded answers to all questions such that higher values reflect stronger religiosity and then used principal component analysis to construct a standardized index.

Previous works proposed numerous ways of assessing institutional distrust from the GSS (Cook & Gronke, 2005). For instance, a two-factor solution to measure trust towards institutions distinguishes between the institutions of order (executive branch, congress, justice system, military, organized religion, major companies) and institutions of opposition (press, labour unions, television, congress) (Cook & Gronke, 2001). An alternative is to measure trust in governmental institutions by focusing only on congress, the executive branch, and the justice system, yet this only evaluates the political dimension of the value (Brehm & Rahn, 1997). For achieving a variable that reveals individuals’ level of disapproval of institutions in general, without reference to any specific one, we generated a generalized institutional distrust variable using all institutions available in the questionnaire. In GSS, presented with 13 institutions^1^, individuals are asked to report their level of confidence in each using a 3-point scale: *“As far as the people running these institutions are concerned*,* would you say you have a great deal of confidence*,* only some confidence*,* or hardly any confidence at all in them?”* Using answers to all 13 institutions for principal component analysis, we construct a standardized index where higher values imply greater distrust.

Generalized trust is measured through a question taken from Rosenberg’s “faith in people” scale (1956): *“Generally speaking*,* would you say that most people are trusted or that you can’t be too careful in dealing with people?”* (Sturgis & Smith, 2010). This is frequently employed as a measurement of generalized trust in social sciences. The answers *“most people can be trusted”* corresponds to a high level of generalized trust, *“can’t be too careful”* implies low, and *“depends”* is a medium level of trust.

The strength of family ties is captured through the reported frequency of an individual’s contact with family members through the question of *“how often you do the following things… Spend a social evening with relatives?”* It differs from alternative measures such as self-reported importance of family since it incorporates geographic proximity to family members. Unlike attitudinal items (e.g., ‘importance of family’), this behavioural proxy captures geographic proximity to relatives—tapping lived kinship intensity, though it may also partially reflect ethnic spatial clustering in the U.S. host context. Therefore, results for family ties should be interpreted with this measurement caveat in mind. Respondents’ answers to the question span from 1 to 7, one corresponding to *“Almost every day”* and seven corresponding to *“Never”.* We recoded them reversely so that higher values correspond to stronger family ties.

The following equation describes the first stage regressions to estimate the inherited values:

1 $${V_{{\mathrm{ict}}}}\,=\,{\beta _0}\,+\,{\beta _{\mathrm{1}}}{D_{\mathrm{c}}}+{\beta _{\mathrm{2}}}{{\mathbf{X}}_{{\mathrm{ict}}}}\,+\,{\varepsilon _{{\mathrm{ict}}}}$$

where the value measure *V* of individual i in year t (whose country-of-origin is c) is regressed on a set of dummy variables indicative of the respondent’s family, *D*_c_, and on socioeconomic characteristics such as age group, sex, educational attainment, social class, employment status, religion, region of interview and generation of immigration. The coefficients for the country-of-origin dummy variables, β_1_, capture the inherited component of culture and are used at the second stage (country level) analysis as the predictors of SDT. The model is identified by omitting one country dummy, Denmark. We select Denmark as the omitted category because it has consistently adequate GSS ancestry coverage across waves and sits near the upper–middle of the distribution for several value indices, making it a transparent benchmark in the SDT literature. Importantly, the choice of reference country does not affect our substantive results: with main effects for values and female education included, the interaction estimates – our main focus – are invariant to re-labelling the base category.

### Validity Checks

Before proceeding to the second stage, we perform two validity checks to enhance the credibility of our inherited value measures. The first validity check examines whether the values we reconstruct from U.S. immigrant descendants (by the ancestral country of origin) align with the contemporary values reported by residents currently living in those origin countries. In other words, we test whether inherited values serve as a valid proxy for contemporary orientations at the national level. To do so, we compare inherited values by ancestral country of origin at time t to those of individuals residing in sampled countries at time t. For a geographical coverage similar to our sampled countries, we retrieve contemporaneous values of residents from the Joint EVS/WVS 2017–2021 dataset (EVS/WVS, 2021). This cross-sectional dataset contains many value questions addressed to individuals residing in 81 territories. Although the most recent data for our sample corresponds to 2017, it is sufficiently close to 2010 to perform a series of basic macro-level comparisons. The EVS/WVS coverage largely overlaps with our sample, with only two omissions (Belgium and Ireland).

The EVS/WVS and GSS questionnaires are comparable in terms of the items measuring the levels of the chosen five values. The specific questions of the Joint EVS/WVS dataset, which we exploit for composing country averages of values, are as follows:

- Gender egalitarianism is measured using the level of agreement to the following 4 statements: *“When a mother works for pay*,* the children suffer” “When jobs are scarce*,* men have more right to a job than women” “On the whole*,* men make better political leaders than women do” “On the whole*,* men make better business executives than women do”.* We recoded the answers so that higher values imply higher gender egalitarianism and then used principal component analysis to construct a standardized index.
- The level of religiosity is assessed using answers to 4 questions: *“How much confidence you have in churches?” “How often do you attend religious services these days?” “How important is religion in your life?” “How important is God in your life?”* While the former two include the institutional dimension of religiosity, the latter two embrace religious beliefs. We recoded the answers such that higher values imply stronger religiosity, then ran a principal component analysis to construct a standardized index.
- The level of institutional distrust is evaluated using a question assessing confidence in various institutions: “*for each item listed*,* how much confidence you have in them*,* is it a great deal*,* quite a lot*,* not very much or none at all?”* The list includes 15 institutions, but to enhance comparability with our main institutional distrust variable, we only exploit the institutions available in the GSS: church, armed forces, press, labour unions, parliament, major companies, and justice system. We used principal component analysis to construct a standardized index in which higher values imply higher institutional distrust.
- Generalized trust is measured using *“Generally speaking*,* would you say that most people can be trusted or that you need to be very careful in dealing with people?”* The answers are recoded on a 3-point scale, *“Most people can be trusted”* as high generalized trust, *“Don’t know”* as intermediate, and *“Can’t be too careful”* as low generalized trust.
- Unfortunately, the questionnaire lacked an item for measuring the frequency of family meetings; hence, we used self-reported importance of family assessed through the question, *“how important is family in your life?”* We recoded the answers such that higher values imply stronger family ties.

**Fig. 2:**
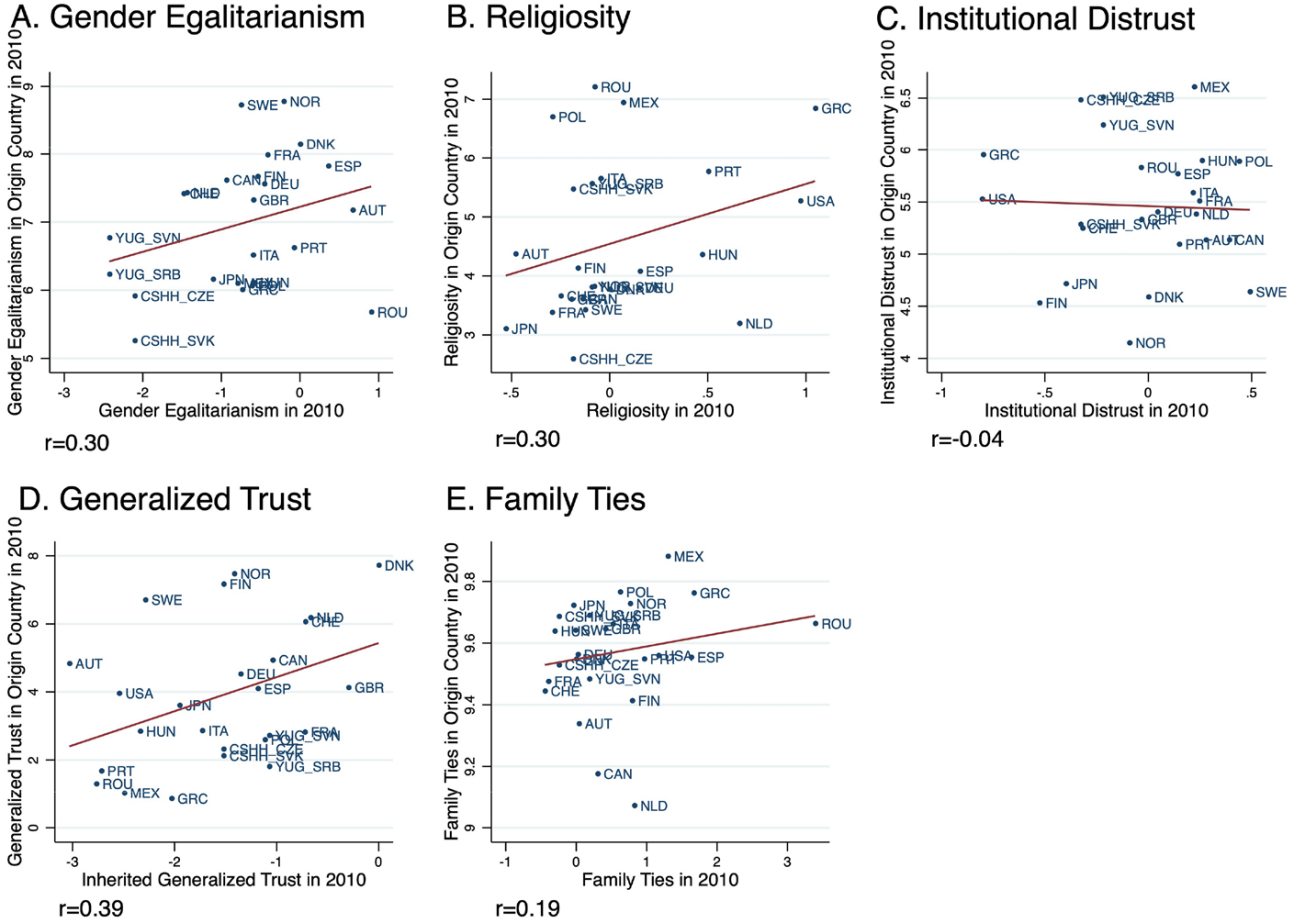
Correlation between inherited and contemporary values in origin countries

Panels A-E of Fig. 2 plots the bivariate relationships between country-level estimations of inherited values in 2010 derived from GSS and country averages of values retrieved from EVS-WVS. Although there is an anticipated measurement error due to question variations and the time difference between the two surveys, the plots show a meaningful consistency when looked together. Four out of five plots demonstrate a positive relationship, and among those, the highest correlation is observed in generalized trust with 0.39, followed by gender egalitarianism (*r* = 0.30), religiosity (*r* = 0.30), and family ties (*r* = 0.19). High correlations imply that the values are strongly transmitted across generations. Differently, institutional distrust showed an extremely low and negative correlation (*r*=-0.04). This relative volatility suggests that distrust is more sensitive to survey context and historical period than other dimensions. Therefore, the analyses taking this specific value as the independent variable will be approached with caution and considered tentative.

The second validity check concerns the relevance of cultural values in explaining demographic behaviours. At the macro level, we expect country-level differences in values to explain macro-demographic changes. As micro-level foundations, individuals are crucial components of macro-level processes. Under a macro–micro–macro logic (Coleman, 1986), the same value dimensions should shape individual partnership and fertility choices, whose aggregation reproduces country patterns (Billari, 2015). Accordingly, this validity check aims to observe a causal link between individual-level demographic behaviours and values. Hence, a micro-level analysis is performed using a question in GSS that evaluates agreement with cohabitation without the intention of getting married through a 5-point scale: *“Do you agree or disagree: is alright for a couple to live together without intending to get married”.* First, we recoded the answers so that the higher values imply higher preferences for cohabitation. Then, we regressed cohabitation preferences on values for the sample of immigrants’ descendants of 2nd, 3rd, and 4th generations. All regressions control for individual-level characteristics and are estimated using ordered probit models^2^ The results are reported in Table 2.

**Table 2:** Impact of values on agreement with cohabitation

Agreement with cohabitation	(1)	(2)	(3)	(4)	(5)
Gender Egalitarianism	0.26***				
	(0.03)				
Religiosity		− 0.27***			
		(0.03)			
Institutional Distrust			− 0.01		
			(0.03)		
Generalized Trust				0.01	
				(0.01)	
Family Ties					− 0.02**
					(0.01)

Observations	800	628	1017	1139	1417
Individual controls	Yes	Yes	Yes	Yes	Yes
Pseudo-R2	0.139	0.148	0.0990	0.0882	0.0879

Robust standard errors in parentheses *p < 0.1; **p < 0.05; ***p < 0.01

**Table 3:** Impact of values on agreement with pre-marital sex

Agreement with Pre-marital Sex	(1)	(2)	(3)	(4)	(5)
Gender Egalitarianism	0.17***				
	(0.01)				
Religiosity		− 0.33***			
		(0.01)			
Institutional Distrust			0.01***		
			(0.00)		
Generalized Trust				0.00	
				(0.00)	
Family Ties					− 0.02***
					(0.00)

Observations	6846	4074	9326	9211	15,715
Individual controls	Yes	Yes	Yes	Yes	Yes
Pseudo-R2	0.108	0.166	0.0774	0.0830	0.0827

Robust standard errors in parentheses *p < 0.1; **p < 0.05; ***p < 0.01

The results of regressions are mostly consistent with the expectations of this validity check. The estimations for three out of five models provide statistically significant effects in the anticipated direction: gender egalitarianism, religiosity, and family ties. Also, these values remain robust when the same validity check is performed using a secondary dependent value: opinions regarding pre-marital sex (Table 3). Thus, they provide solid evidence regarding the role of micro-foundations on macro-level demographic change. However, the models for generalized trust and institutional distrust demonstrate insignificant effects on the opinions regarding cohabitation. In the additional validity done utilizing pre-marital sex, the impact becomes significant in the anticipated direction for institutional distrust. However, insignificance persists for generalized trust.

Overall, the validity checks have largely confirmed the transmission of values across generations and the enduring influence of inherited values on contemporary demographic behaviours, with gender egalitarianism, religiosity, and family ties showing robust significant alignment with anticipated trends. These results underscore the potent role of such values as stable predictors within the framework of macro-level demographic shifts. However, generalized trust and institutional distrust present a more complex picture. Generalized trust, while showing a positive correlation in the first validity check, did not pass the second, suggesting that its role in influencing demographic behaviours such as cohabitation preferences is not as pronounced. This indicates a possible decoupling of generalized trust from certain demographic behaviours, calling for a nuanced analysis of this value’s role within the demographic outcomes. Differently, institutional distrust’s low negative correlation in the first validity check points to its relative volatility and sensitivity to current socio-political climates, marking it as a variable that requires cautious interpretation within the context of intergenerational value transmission. Despite failing to pass the first validity check, the variable institutional trust will still be employed for exploratory purposes; however, any results stemming from this variable will be interpreted within the context of its recognized constraints and regarded as tentative.

### Second Stage: Country-Level Data and Analyses

In the second stage, the outcome is the share of births outside marriage (OECD Family Database), observed at two anchors (1960, 2010) to capture long‑run change. We combine this outcome with country-level data on female tertiary education for 1960 and 2010. The resulting sample covers fkls countries, including Austria, Belgium, Canada, Switzerland, Czechia, Germany, Denmark, Spain, Finland, France, Great Britain, Greece, Hungary, Ireland, Italy, Japan, Mexico, Netherlands, Norway, Poland, Portugal, Romania, Serbia. When considered a group, these countries constitute the majority of the member countries of the Organization of Economic Co-Operation and Development (OECD); thus, they share various contextual factors.

A critical problem regarding educational attainment and school enrolment data is the lack of detailed information for earlier periods. Therefore, we retrieved the percentage of tertiary schooling attained by the female population from Barro-Lee Datasets on Long-Run Enrolment Ratios and Educational Attainment (Lee & Lee, 2016). This dataset contains estimated school enrolment ratios from 1820 to 2010 and estimated educational attainment for total, female, and male populations from 1870 to 2010. The estimates are available for every five years in 111 countries, providing extensive coverage in time and geography.

Unfortunately, there is no universally accepted indicator for measuring SDT levels. Indeed, since the foundation of the theory in 1986, a consensus on the measurement of SDT levels has not been reached in the literature. Various scholars have attempted to operationalize the concept using individual indices that capture different behavioural aspects of SDT, encompassing factors like non-marital births, cohabitation, age at childbirth and marriage, total fertility rates, and divorce rates (Brzozowska, 2021; Bystrov, 2014; Lesthaeghe & Neidert, 2006a, b; Liefbroer et al., 2015; Potârcă et al., 2013; van de Kaa, 2001). Additionally, there have been efforts to devise composite or summary indices (Lesthaeghe & Neidert, 2009; Sobotka, 2008; Valkonen et al., 2008). While these composite indices are valuable for providing an overarching perspective and are easier to interpret than analysing individual indicators, constructing them poorly can lead to information loss and reduced accuracy. Therefore, we opt to utilize single behavioural indicators of SDT.

As an indicator of a country’s SDT levels, we have selected the proportion of births outside marriage, which we sourced from the OECD Family Database (OECD, 2021). The choice of the indicator is based on three reasons: non-marital births’ frequent citation in theoretical and empirical works of SDT, the availability of long-term past data, and, most importantly, the relevance of SDT theory in explaining cross-country differences in non-marital birth shares. The increasing disconnection between childbearing and marriage has been one of the most remarkable changes in nuptiality regimes over the past 50 years (Sobotka & Toulemon, 2008). Its steep increase began during the early 1970s in Northern Europe and spread over most European countries, America, Australia, and Oceania. SDT framework interpreted this pattern as a progression driven mainly by value shifts that free individuals from conforming to conventional family forms (Lesthaeghe, 2010; van de Kaa, 1987). Several works have criticized this perspective due to its inability to explain “the pattern of disadvantage”, meaning the negative educational and socioeconomic gradient of childbearing outside marriage, observed primarily in Latin American countries (Esteve et al., 2012) as well as in some parts of Europe (Perelli-Harris et al., 2010) and United States (Upchurch et al., 2002). However, in their study comparing both perspectives’ ability to explain non-marital birth shares, Lappegård et al. (2014) showed that the SDT framework is essential for understanding the cross-countries disparities. However, the pattern of disadvantage hypothesis is more relevant for within-country comparisons observing variation between individuals or subnational regions.

The impact of long-standing inherited values on the SDT can be represented with the following linear model:

2 $$ SDT_{{{\mathrm{ct}}}} = {\text{ }}\alpha _{0} + {\text{ }}\alpha _{{\mathrm{1}}} \,\widehat{{\beta _{1} }}_{{{\mathrm{ct}}}} \, \times E_{{{\mathrm{ct}}}} + {\text{ }}\Sigma ^{{\mathrm{k}}} \alpha _{{\mathrm{k}}} X_{{{\mathrm{kct}}}} + F_{{\mathrm{c}}} + F_{{\mathrm{t}}} + {\text{ }}\eta _{{{\mathrm{ct}}}} $$

Here *SDT*_ct_ is non-marital birth rates of country c in year t, $\widehat{{\beta _{1} }}_{{{\mathrm{ct}}}}$_ct_ is the average level of a given value estimated at the 1st stage, *E*_ct_ is the average tertiary education level of females in country c in year t, the *X* are time-varying social, economic and institutional covariates and η_ct_ is the error term. Eventually, the coefficient linking inherited values to the non-marital birth rates, namely α_1_, reflects the importance of specific long-standing values over the progress of SDT.

## Results

### Variation of Values Over 1960 and 2010

**Fig. 3:**
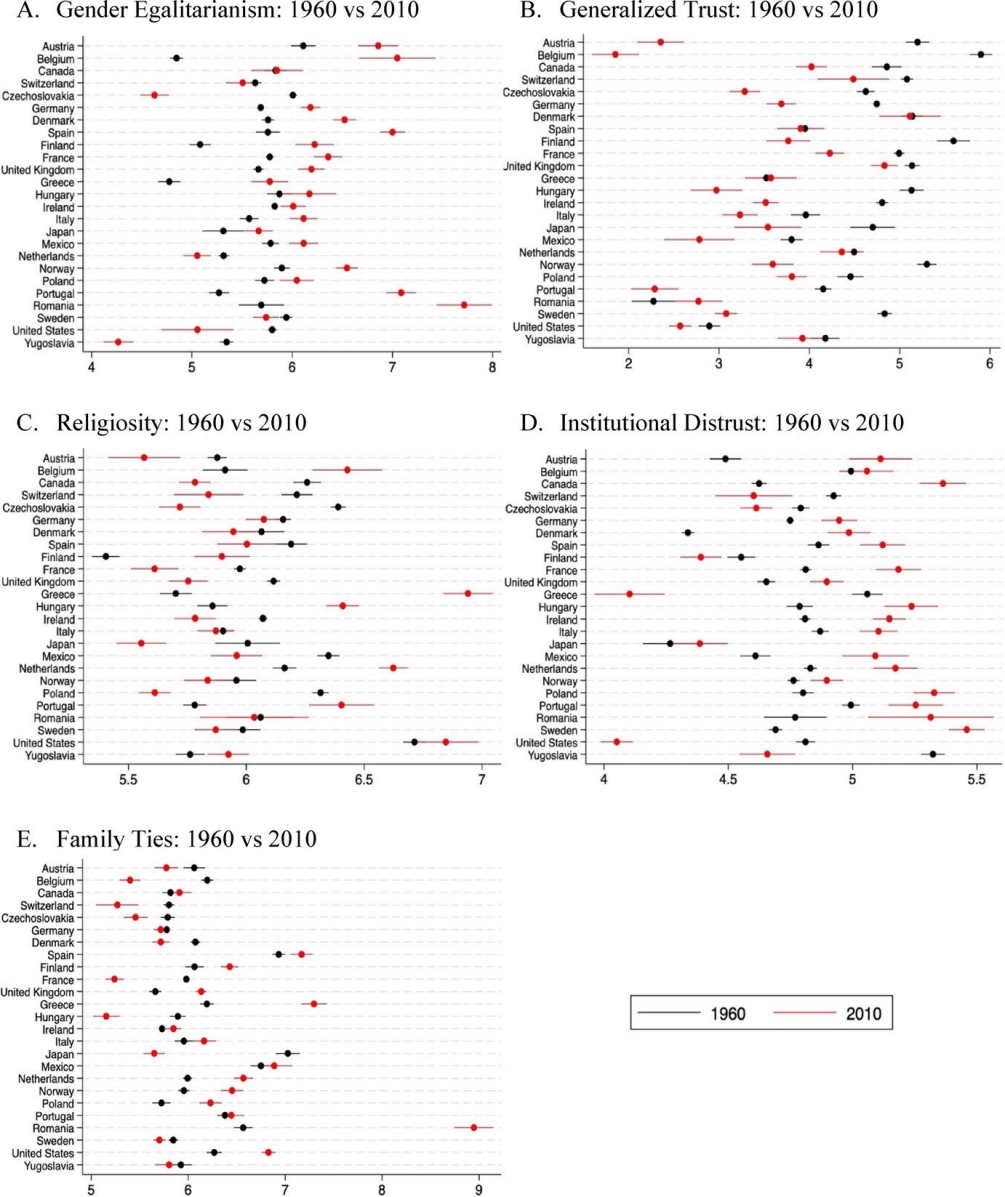
Variation of inherited values across 1960 and 2010, as reconstructed from U.S. GSS immigrant-descendant data

Before delving into the results of macro-level analyses, it’s crucial to understand how values have evolved over time. The exploration of country-level value changes will provide a comprehensive view of the sampled period and serves as the descriptive foundation for our empirical strategy. Panels A-E of Fig. 3 plots for country averages with confidence intervals for five values in 1960 and 2010. These averages are not simply descriptive statistics but represent the inherited value measures derived through our epidemiological reconstruction. In other words, Fig. 3 illustrates how the cohort-based anchoring of inherited values translates into observable cross-country variation at two distant points in time. This step links the methodological design of Stage 1 to the macro-level analyses of Stage 2.

For example, gender egalitarianism experienced a big jump in the sampled countries over the 50-year period. In 1960, gender-egalitarian values were mostly within the range of 5–6, but by 2010, they had shifted beyond 6 (Panel A of Fig. 3). Another notable change occurred in generalized trust, which substantially decreased over the years. In 1960, most countries had values between 4 and 5, while in the subsequent 50 years, they moved within the range of 3–4 (Panel B of Fig. 3). These distinct patterns of change are important to consider when interpreting the macro-level analysis, as significant overall increases or decreases could potentially impact the main effects of inherited values. However, our focus lies on the interaction of inherited values with female education; therefore, main effects are reported for completeness, but interpretation centres on the interaction terms that match our theory.

### Impact of Inherited Values on Second Demographic Transition

Table 4 reports coefficients for 10 models estimating the impact of specific inherited values on the share of non-marital births in 1960 and 2010. For each value, the first model demonstrates the bivariate relationship, while the second includes fixed effects (FE). All models control for macro-level variables of median age, unemployment rate, and log GDP per capita. The focus of our analysis is the interaction between inherited values and female education, which allows us to delve deeper into the nuanced mechanisms driving the second demographic transition. Inherited values serve as a foundational backdrop that shapes individual attitudes and behaviours, while the level of female education functions as a catalyst that either amplifies or mitigates these values’ influence on demographic trends. We hypothesize that higher levels of female education, as the key structural enabler, may intensify the effects of inherited values related to non-conformism, self-fulfilment, and individualization, thereby accelerating the adoption of alternative family arrangements and divergent fertility patterns.

Overall, three out of five inherited values’ interaction with female education produce statistically significant effects in the expected direction; these include gender egalitarianism, institutional distrust, and generalized trust. We observe that the estimate’s magnitude and statistical significance increase across specifications without and with fixed effects for each of these three values. For the case of inherited gender egalitarianism, both models indicate large positive effects (b = 0.75 without FE, b = 0.89 with FE), and the significance level increases from *p* < 0.05 to *p* < 0.01 across models. This finding is consistent with the notion that education expands women’s autonomy and makes inherited egalitarian values actionable, thereby translating into demographic change.

Greater institutional distrust also has a sizeable significant effect on non-marital births in the expected positive direction (b = 0.71 without FE, b = 0.83 with FE), and the strength of significance increases from *p* < 0.1 to *p* < 0.05 across models. This can be read as, higher education reducing dependence on formal authority (state, church, registry) by raising economic security and legal literacy, thus, in settings with inherited scepticism toward institutions, this structural independence increases the attractiveness of non-institutionalized unions and non-marital childbearing. Also, we made further analyses using different types of institutional distrust, precisely the two-factor measurement suggested by Cook and Gronke (2001) and distrust of only governmental institutions suggested by Brehm and Rahn (1997). The results remain robust across all types of institutional distrust and are reported in Table 5 in the Appendix. However, the validity checks indicate a low negative correlation for institutional distrust in the first check, suggesting its relative volatility and sensitivity to socio-political climates. Consequently, results related to this variable should be regarded as tentative and further analyses using different data or methodologies are needed.

Moreover, generalized trust generates significant positive effects (b = 0.31 without FE, b = 0.42 with FE), and the significance level rises from *p* < 0.1 to *p* < 0.01 across models. The validity checks for this value present a complex picture. Although generalized trust passed the first validity check, indicating persistence over the years, it failed to pass the second validity check. This failure suggests a possible decoupling from divergent demographic behaviours. However, the robustness of the significant positive effect indicates that generalized trust does impact non-marital birth rates.

In this case, structural enhancements as female education fosters the activation of inherited trust for instance by expanding women’s labour-market participation and access to services; where generalized trust is high, the perceived risks and coordination costs of cohabitation and non-marital parenting are lower, making alternative family forms more viable. To strengthen this argument within the theoretical framework of second demographic transition, further analysis should be conducted using additional SDT indicators as more data becomes available.

In the case of inherited family ties, we observe that its interaction with education is in the expected direction for both models (b=-0.14 without FE, b=-0.39 with FE), but the statistical significance is present only when the fixed effects are included. Here, structural enhancement’s enabling effects are constrained by inherited strong ties: intergenerational support is channelled toward marriage and sequencing norms, and family-based insurance substitutes for welfare/market solutions, dampening the translation into non-conventional family forms like non-marital childbearing.

Though religiosity produces results in the expected negative direction, the estimates of interaction with female education remain statistically insignificant across models with and without fixed effects. This pattern indicates that educational expansion as a structural enhancement does not systematically activate or suppress inherited religiosity in shaping non-marital childbearing. Given the bi-dimensional construction of our religiosity index (belief vs. institutional attachment/participation), education may amplify one component while dampening the other, yielding no net interaction at the macro level.

Taken together, the models show female education operates as a structural “switch” that activates or dampens inherited cultural endowments: it amplifies autonomy-oriented traits (gender egalitarianism, generalized trust, institutional distrust) into higher non-marital births, while strong family ties mute this translation. Religiosity shows no consistent moderation, likely because its belief and institutional components offset each other. Overall, cross-country SDT variation reflects not education or culture alone, but their interaction—how widening educational opportunities foster or impede what is already culturally inherited.

**Table 4:** Non-marital births and inherited values

Non-Marital Birth Rates	(1)	(2)	(3)	(4)	(5)	(6)	(7)	(8)	(9)	(10)
Gender Egalitarianism	− 8.40	− 14.45***								
	(5.49)	(4.25)								
Religiosity			4.77	13.20						
			(11.14)	(13.66)						
Institutional Distrust					− 10.70	− 17.25				
					(9.75)	(15.76)				
Generalized Trust							− 8.91**	− 12.71***		
							(3.63)	(2.46)		
Family Ties									6.10*	23.50***
									(3.47)	(7.99)
Female Education	1.44***	1.77***	0.77**	1.27***	0.79**	1.15***	1.05***	1.56***	0.80**	1.23***
	(0.35)	(0.31)	(0.35)	(0.29)	(0.31)	(0.26)	(0.35)	(0.34)	(0.34)	(0.28)
Gender Egalitarianism	0.75**	0.89***								
× Female Education	(0.28)	(0.21)								
Religiosity			− 0.14	− 0.39						
× Female Education			(0.42)	(0.30)						
Institutional Distrust					0.71*	0.83**				
× Female Education					(0.37)	(0.35)				
Generalized Trust							0.31*	0.42***		
× Female Education							(0.16)	(0.13)		
Family Ties									− 0.27	− 0.75***
× Female Education									(0.24)	(0.20)

Observations	44	44	44	44	44	44	44	44	44	44
Country FE	No	Yes	No	Yes	No	Yes	No	Yes	No	Yes
Controls	Yes	Yes	Yes	Yes	Yes	Yes	Yes	Yes	Yes	Yes
R2	0.59	0.59	0.49	0.49	0.56	0.56	0.56	0.56	0.51	0.51

Robust standard errors in parentheses *p < 0.1; **p < 0.05; ***p < 0.01

## Conclusion

This study aims to provide insights on why the behavioural outcomes of the second demographic transition have unfolded so unevenly across otherwise similar Western societies. We argued that persistent cross‑national differences are not only a matter of when the post‑war ideational shift reached a given society, but also of how that shift interacted with long‑standing, inherited cultural values. Using an epidemiological strategy to proxy the inherited components of five values – gender egalitarianism, religiosity, institutional distrust, generalized trust, and family ties – and combining these measures with macro indicators at two anchors (1960 and 2010), we analyse how, in interaction with major structural change (female tertiary education expansion) they effect one of one core SDT outcome, namely the share of births outside marriage. Specifically, we have focused on the interaction between inherited values and levels of female tertiary education, as this interaction may amplify the effects of values related to non-conformism, self-fulfilment, and individualization. Consequently, it could accelerate the adoption of alternative family arrangements and divergent fertility patterns.

Gender egalitarianism, institutional distrust, and generalized trust were found to be significant predictors, with their effects becoming more pronounced when fixed effects were included in the models. This can be interpreted as, with the broad expansion of education that has taken place across all Western countries after the IIWW, the SDT spreads much faster in those societies where inherited gender egalitarianism are strong. Interpreted differently, the effect of education on the spread of SDT is more powerful, where inherited values have a strong component of gender egalitarianism.

In contrast, inherited family ties showed a significant negative impact in the fixed effects model, indicating that stronger family ties are associated with lower rates of non-marital births when controlling for fixed effects. This is to say, with the broad expansion of education that has taken place across all Western countries after the IIWW, the SDT spreads much slower in those societies where inherited family ties are strong. Interpreted differently, the effect of education on the spread of SDT is less powerful, where inherited values have a strong component of family ties. Our study does not suggest that SDT will not happen in these countries but offers a tangible explanation for why it has spread more slowly.

Taken together, autonomy‑oriented endowments (egalitarianism, generalized trust, institutional skepticism) accelerate the spread of SDT behaviours when education expands; familism slows it. This perspective complements rather than rejects the classic SDT emphasis on a broad post‑materialist value shift. Our contribution is to show that the speed and form of diffusion hinge on which deep values are already in place when structural preconditions – most notably women’s education – change. Calling for an SDT framework that explicitly models culture–structure interactions, the findings imply that studying the second demographic transition necessitates a finer-tuned cultural approach acknowledging the importance of enduring local values. By pointing towards specific value differences transmitted across generations, such an approach may bring more rigor to explaining persistent variations in demographic trends across societies.

Methodologically, we demonstrate that epidemiological approach can recover time‑anchored proxies of inherited values in settings where historical survey data are unavailable. Two validation exercises support the credibility of these measures: cross‑sectional alignment with EVS/WVS country profiles (especially for generalized trust and gender egalitarianism), and micro‑level linkages between values and attitudes toward cohabitation and premarital sex within the GSS immigrant‑descendant sample (notably for gender egalitarianism, religiosity, and family ties).

We also recognize important limitations. Our empirical scope is deliberately narrow: we analyze a single SDT outcome – the share of births outside marriage – chosen for its historical depth, cross-national comparability, and close conceptual link to the SDT’s ideational mechanism (the decoupling of marriage and childbearing). Other SDT behaviours (cohabitation, divorce, fertility postponement) lie beyond the present analysis and are candidates for replication, contingent on data availability. Further, while we cannot directly observe the full stability of intergenerational transmission within each origin country, our interpretation is anchored in the established transmission literature and in our two validation steps, which indicate persistence sufficient for proxy use. Lastly, our proxies rely on respondents in a single host country (the United States), which may entail selection and adaptation biases despite controls and generation restrictions included in our analyses.

## Appendix

See Table 5.

**Table 5:** Non-Marital births and inherited institutional Distrust

Non-marital Birth Rates	(1)	(2)	(3)	(4)	(5)	(6)	(7)	(8)
General Institutional	− 10.70	− 17.25						
Distrust	(9.75)	(15.76)						
Institutional Distrust			− 16.30	− 31.35*				
(Order)			(10.00)	(18.07)				
Institutional Distrust					− 18.80**	− 23.73**		
(Opposition)					(8.57)	(8.88)		
Institutional Distrust							− 22.02	− 33.99*
(Governmental)							(12.91)	(16.82)
Female Education	0.79**	1.15***	0.95***	1.34***	1.11***	1.48***	0.87**	1.29***
	(0.31)	(0.26)	(0.32)	(0.23)	(0.38)	(0.34)	(0.34)	(0.29)
General Institutional	0.71*	0.83**						
Distrust × Fem Ed	(0.37)	(0.35)						
Institutional Distrust			0.81**	1.07***				
(Order) × Fem Ed			(0.36)	(0.29)				
Institutional Distrust					1.04**	1.07***		
(Oppos.) × Fem Ed					(0.47)	(0.26)		
Institutional Distrust							0.92**	1.14***
(Gov.) × Fem Ed							(0.43)	(0.30)
Observations	44	44	44	44	44	44	44	44
Country FE	No	Yes	No	Yes	No	Yes	No	Yes
Controls	Yes	Yes	Yes	Yes	Yes	Yes	Yes	Yes
R2	0.56	0.59	0.57	0.57	0.61	0.61	0.57	0.57

Robust standard errors in parentheses *p < 0.1; **p < 0.05; ***p < 0.01

## Data Availability

The data that support the findings of this study are available from the corresponding author upon reasonable request.

## Footnotes

1 The presented institutions include banks and financial institutions, major companies, organized religion, education, executive branch of the federal government, organized labor, press, medicine, TV, US supreme court, scientific community, congress and military.

2 Controlled individual level characteristics include age, age squared, sex, educational attainment, income, employment status, religion, generation of immigration, region of interview.
